# Supplementary material for: Within My Walls, I Escape Being Underestimated: A Systematic Review and Thematic Synthesis of Stigma and Help-Seeking in Dementia
Source: Behav Sci (Basel). 2025 Jun 3;15(6):774. doi: 10.3390/bs15060774 (PMC12189192; doi:10.3390/bs15060774)
Supplement: Supplementary file 1 [file behavsci-15-00774-s001.zip › behavsci-3612612-supplementary.pdf]

GRADE-CERQual Summary of Qualitative Findings

Review Findings	Methodological Limitations	Coherence	Adequacy of Data	Relevance	Confidence Level	References
**Reluctance to Share One's Condition and Risk of Labelling.** The perception of dementia as incurable exacerbates psychological distress and discourages help-seeking, while fear of labeling contributes to social exclusion and emotional withdrawal.	**Minor**<br><br>It is not possible to determine whether appropriate ethical approval was obtained.	**No/very minor concerns**	**Minor**<br><br>It is unclear whether participants had a formal diagnosis of dementia, and the perspective of individuals living with dementia regarding stigma is not adequately distinguished in the findings.	**No/very minor concerns**	**Moderate**<br><br>No/very minor concerns regarding coherence, and no/very minor concerns regarding relevance, minor concerns regarding methodological limitations and Minor concerns regarding adequacy.	Biswas et al., 2022; Cheung et al., 2022; Lian et al., 2017; Mitchell et al., 2020; Molvik et al., 2024; Pavković et al., 2025; Toms et al., 2015; Zhang et al., 2020
**From Public Perception to Internalization or Rejection of Stigmatizing Beliefs.** Perceived stigma, rooted in societal marginalization, hinders dementia recognition and support-seeking. Individuals often internalize these attitudes, leading to self-stigma and diminished self-worth, though some actively resist such internalization to avoid pity or condescension.	**Minor**<br><br>The relationship between researchers and participants was not sufficiently addressed, limiting the assessment of potential influence on data collection and interpretation.<br><br>It is not possible to determine whether appropriate ethical approval was obtained.	**No/very minor concerns**	**Minor**<br><br>The experience of people with dementia is not adequately represented.	**No/very minor concerns**	**Moderate**<br><br>No/very minor concerns regarding coherence, no/very minor concerns regarding relevance, minor concerns regarding methodological limitations, minor concerns regarding adequacy.	Biswas et al., 2022<br>Mitchell et al., 2020<br>Pavković et al., 2025<br>Strier and Werner, 2015

**How Family and Community Shape the Experience of Seeking-help of People with dementia.** Familial and community stigma, often based on misconceptions, delays help-seeking and increases isolation for people with dementia, though supportive family environments, linked to No/very minor concernser education, can mitigate these effects and aid coping.	**Minor** It is not possible to determine whether appropriate ethical approval was obtained.	**Minor** Some data are ambiguous, as it is unclear whether they exclusively reflect the perspective of the person living with dementia.	**Minor** The experience of people with dementia is not adequately represented.	**No/very minor concerns**	**Moderate** No/very minor regarding relevance, minor concerns regarding methodological limitations, minor concerns regarding coherence and minor concerns regarding adequacy.	Hurzuk et al., 2022 Lian et al., 2017 Mitchell et al., 2020 Willis et al., 2020 Zhang et al., 2020
**Professional Attitudes and Eligibility Processes in Dementia Services.** Healthcare professionals' limited knowledge and stigmatizing attitudes, including infantilization and nihilism, obstruct dementia care access and trust. Systemic inequities and offensive eligibility processes further	**Minor** The relationship between researchers and participants was not sufficiently addressed, limiting the assessment of potential influence on data collection and interpretation. It is not possible to determine whether appropriate	**Minor** Some data are ambiguous, as it is unclear whether they exclusively reflect the perspective of the person living with dementia.	**Minor** The experience of people with dementia is not adequately represented.	**No/very minor concerns**	**Moderate** No/very minor concerns regarding relevance, minor concerns regarding methodological limitations, minor concerns regarding coherence, minor concerns regarding adequacy.	Dooley et al., 2025 Farhana et al., 2023 Garrett et al., 2024 Hurzuk et al., 2022 Mitchell et al., 2020 Pavković et al., 2025 Strier and Werner, 2015 Zhang, Clarke, and Rhynas, 2020

alienate patients, reinforcing treatment stigma.	ethical approval was obtained.					
**Stigma Stems from Lack of Awareness and Knowledge of Dementia.** Stigma surrounding dementia largely arises from widespread societal ignorance and stereotypical portrayals, which depict individuals with dementia as incompetent or tragic, reinforced by dehumanizing media language. This fosters fear and reluctance to seek help, exacerbated by inadequate public education and representation in care planning.	**Minor** The relationship between researchers and participants was not sufficiently addressed, limiting the assessment of potential influence on data collection and interpretation. It is not possible to determine whether appropriate ethical approval was obtained.	**No/very minor concerns**	**Minor** The experience of people with dementia is not adequately represented.	**No/very minor concerns**	**No/very minor concerns** No/very minor concerns regarding coherence, and no/very minor concerns regarding relevance, minor concerns regarding methodological limitations, minor concerns regarding adequacy.	Dooley et al., 2025; Farhana et al., 2023; Hurzuk et al., 2022; Lian et al., 2017; Martin et al., 2013; Mitchell et al., 2020; Strier and Werner, 2015; Walker et al., 2023; Zhang et al., 2020
**The Impact of Psychological Decline, Isolation, and Loss of Autonomy on Help-Seeking.** As dementia advances, individuals experience fear of memory loss, loss of independence, and emotional distress, which impair their ability to seek support and lead to social withdrawal and	**Minor** The relationship between researchers and participants was not sufficiently addressed, limiting the assessment of potential influence on data collection and interpretation. It is not possible to determine whether appropriate	**No/very minor concerns**	**Minor** The experience of people with dementia is not adequately represented.	**No/very minor concerns**	**No/very minor concerns** No/very minor concerns regarding coherence, and no/very minor concerns regarding relevance, minor concerns regarding methodological limitations, minor concerns regarding adequacy.	Carter et al., 2024 Cheung et al., 2022 Dooley et al., 2025 Hurzuk et al., 2022 Martin et al., 2013 Mitchell et al., 2020 Molvik et al., 2024 Pavković et al., 2025 Toms et al., 2015 Walker et al., 2023

loneliness. However, diagnosis can also provide relief and promote self-directed coping and help-seeking.	ethical approval was obtained.					
**A Gap in Accessible and Supportive Services.** A significant barrier for people with dementia is the widespread lack of awareness about available services, compounded by cultural, informational, and systemic obstacles, resulting in delayed diagnosis and fragmented care. Service inadequacies, workforce shortages, physical inaccessibility, and limited non-pharmacological options further restrict effective support and exacerbate distrust in institutional care.	**Minor**<br>The relationship between researchers and participants was not sufficiently addressed, limiting the assessment of potential influence on data collection and interpretation.<br>It is not possible to determine whether appropriate ethical approval was obtained.	**Minor**<br>Some data are ambiguous, as it is unclear whether they exclusively reflect the perspective of the person living with dementia.	**Minor**<br>The experience of people with dementia is not adequately represented.	**No/very minor concerns**	**Moderate**<br>No/very minor concerns regarding relevance, minor concerns regarding methodological limitations, minor concerns regarding coherence, minor concerns regarding adequacy.	Biswas et al., 2022<br>Dooley et al., 2025<br>Farhana et al., 2024<br>Garrett et al., 2024<br>Hurzuk et al., 2022<br>Lian et al., 2017<br>Martin et al., 2013<br>Pavković et al., 2025<br>Strier and Werner, 2015<br>Walker et al., 2023<br>Zhang et al., 2020
**Caregiver Support, Professional Relationships, and Peer Support.** Caregivers play a crucial role in early symptom recognition and initiating help-	**Minor**<br>It is not possible to determine whether appropriate ethical approval was obtained.	**No/very minor concerns**	**No/very minor concerns**	**No/very minor concerns**	**No/very minor concerns**<br>No/very minor concerns regarding coherence, no/very minor concerns regarding	Dooley et al., 2025<br>Garrett et al., 2024<br>Lian et al., 2017<br>Martin et al., 2012<br>Pavković et al., 2025<br>Toms et

seeking, supported by extended family networks. Positive healthcare professional relationships and peer support also enhance engagement and reduce stigma, improving care experiences for people with dementia.					adequacy and no/very minor concerns regarding relevance, minor concerns regarding methodological limitations.	al., 2014<br>Willis et al., 2020
**What Government Can Do to Tackle Dementia.** Governmental policies and public health initiatives, particularly targeted awareness campaigns and improved local healthcare accessibility, are crucial to reducing stigma and promoting early help-seeking in dementia.	**Minor**<br>The relationship between researchers and participants was not sufficiently addressed, limiting the assessment of potential influence on data collection and interpretation.<br>It is not possible to determine whether appropriate ethical approval was obtained.	**Minor**<br>Some data are ambiguous, as it is unclear whether they exclusively reflect the perspective of the person living with dementia.	**Moderate**<br>The experience of people with dementia are not sufficiently represented	**No/very minor concerns**	**Moderate**<br>No/very minor concerns regarding adequacy, minor concerns regarding methodological limitations, minor concerns regarding relevance, moderate concerns regarding adequacy.	Lian et al., 2017<br>Walker et al., 2023

Summary of review findings

Summary of review findings	*GRADE-CERQual assessment of confidence in the evidence*	*Explanation of GRADE-CERQual assessment*	*Studies contributing to the review findings*
Reluctance to Share One's Condition and Risk of Labelling. *The perception of dementia as incurable exacerbates psychological distress and discourages help-seeking, while fear of labeling contributes to social exclusion and emotional withdrawal.*	**Moderate**	No/very minor concerns regarding coherence, and no/very minor concerns regarding relevance, minor concerns regarding methodological limitations and Minor concerns regarding adequacy	Biswas et al., 2022; Cheung et al., 2022; Lian et al., 2017; Mitchell et al., 2020; Molvik et al., 2024; Pavković et al., 2025; Toms et al., 2015; Zhang et al., 2020
From Public Perception to Internalization or Rejection of Stigmatizing Beliefs. *Perceived stigma, rooted in societal marginalization, hinders dementia recognition and support-seeking. Individuals often internalize these attitudes, leading to self-stigma and diminished self-worth, though some actively resist such internalization to avoid pity or condescension.*	**Moderate**	No/very minor concerns regarding coherence, no/very minor concerns regarding relevance, minor concerns regarding methodological limitations, minor concerns regarding adequacy.	Biswas et al., 2022<br>Mitchell et al., 2020<br>Pavković et al., 2025<br>Strier and Werner, 2015
How Family and Community Shape the Experience of Seeking-help of People with dementia. *Familial and community stigma, often based on misconceptions, delays help-seeking and increases isolation for people with dementia, though supportive family environments, linked to education, can mitigate these effects and aid coping.*	**Moderate**	No/very minor regarding relevance, minor concerns regarding methodological limitations, minor concerns regarding coherence and minor concerns regarding adequacy.	Hurzuk et al., 2022<br>Lian et al., 2017<br>Mitchell et al., 2020<br>Willis et al., 2020<br>Zhang et al., 2020
Professional Attitudes and Eligibility Processes in Dementia Services. *Healthcare professionals' limited knowledge and stigmatizing attitudes, including infantilization and nihilism, obstruct dementia care access and trust. Systemic inequities*	**Moderate**	No/very minor concerns regarding relevance, minor concerns regarding methodological limitations, minor concerns regarding coherence, minor concerns regarding adequacy.	Dooley et al., 2025<br>Farhana et al., 2023<br>Garrett et al., 2024<br>Hurzuk et al., 2022<br>Mitchell et al., 2020<br>Pavković et al., 2025<br>Strier and Werner, 2015<br>Zhang, Clarke, and Rhynas, 2020

and offensive eligibility processes further alienate patients, reinforcing treatment stigma.			
Stigma Stems from Lack of Awareness and Knowledge of Dementia. *Stigma surrounding dementia largely arises from widespread societal ignorance and stereotypical portrayals, which depict individuals with dementia as incompetent or tragic, reinforced by dehumanizing media language. This fosters fear and reluctance to seek help, exacerbated by inadequate public education and representation in care planning.*	**No/very minor concerns**	No/very minor concerns regarding coherence, and no/very minor concerns regarding relevance, minor concerns regarding methodological limitations, minor concerns regarding adequacy.	Dooley et al., 2025; Farhana et al., 2023; Hurzuk et al., 2022; Lian et al., 2017; Martin et al., 2013; Mitchell et al., 2020; Strier and Werner, 2015; Walker et al., 2023; Zhang et al., 2020
The Impact of Psychological Decline, Isolation, and Loss of Autonomy on Help-Seeking. *As dementia advances, individuals experience fear of memory loss, loss of independence, and emotional distress, which impair their ability to seek support and lead to social withdrawal and loneliness. However, diagnosis can also provide relief and promote self-directed coping and help-seeking.*	**No/very minor concerns**	No/very minor concerns regarding coherence, and no/very minor concerns regarding relevance, minor concerns regarding methodological limitations, minor concerns regarding adequacy.	Carter et al., 2024<br>Cheung et al., 2022<br>Dooley et al., 2025<br>Hurzuk et al., 2022<br>Martin et al., 2013<br>Mitchell et al., 2020<br>Molvik et al., 2024<br>Pavković et al., 2025<br>Toms et al., 2015<br>Walker et al., 2023
A Gap in Accessible and Supportive Services. *A significant barrier for people with dementia is the widespread lack of awareness about available services, compounded by cultural, informational, and systemic obstacles, resulting in delayed diagnosis and fragmented care. Service inadequacies, workforce shortages, physical inaccessibility, and limited non-*	**Moderate**	No/very minor concerns regarding relevance, minor concerns regarding methodological limitations, minor concerns regarding coherence, minor concerns regarding adequacy.	Biswas et al., 2022<br>Dooley et al., 2025<br>Farhana et al., 2024<br>Garrett et al., 2024<br>Hurzuk et al., 2022<br>Lian et al., 2017<br>Martin et al., 2013<br>Pavković et al., 2025<br>Strier and Werner, 2015<br>Walker et al., 2023<br>Zhang et al., 2020

pharmacological options further restrict effective support and exacerbate distrust in institutional care.			
Caregiver Support, Professional Relationships, and Peer Support. *Caregivers play a crucial role in early symptom recognition and initiating help-seeking, supported by extended family networks. Positive healthcare professional relationships and peer support also enhance engagement and reduce stigma, improving care experiences for people with dementia.*	**No/very minor concerns**	No/very minor concerns regarding coherence, no/very minor concerns regarding adequacy and no/very minor concerns regarding relevance, minor concerns regarding methodological limitations	Dooley et al., 2025<br>Garrett et al., 2024<br>Lian et al., 2017<br>Martin et al., 2012<br>Pavković et al., 2025<br>Toms et al., 2014<br>Willis et al., 2020
What Government Can Do to Tackle Dementia. *Governmental policies and public health initiatives, particularly targeted awareness campaigns and improved local healthcare accessibility, are crucial to reducing stigma and promoting early help-seeking in dementia.*	**Moderate**	No/very minor concerns regarding adequacy, minor concerns regarding methodological limitations, minor concerns regarding relevance, moderate concerns regarding adequacy.	Lian et al., 2017<br>Walker et al., 2023
